# Pay Hospitals or Medical Hotels

**Published:** 1895-04-20

**Authors:** 


### The Hospital

AN INSTITUTIONAL JOURNAL OF

### The Medical Sciences and Hospital Administration,

WITH

AN EXTRA NURSING SUPPLEMENT.

VOL. XVIII.—No. 447.] [APRIL 20, 1895.

### PAY HOSPITALS OR MEDICAL HOTELS ?

WHILST medical men are wrangling among themselves about the "commercialisation" of medicine, outside pretenders to medical knowledge are taking up portions of the medical art in the spirit of modern commerce, and in some instances amassing fortunes thereby. It has long been the practice to advertise what may be called "counter" remedies—powders, pills, mixtures, ointments, and other commodities which may be sold in the shop. Now it is becoming the fashion to advertise "specialisms," medical and surgical; and a considerable number of persons find it more profitable to acquire a little knowledge of specialisms and special operations, and to practise them as unqualified practitioners, than to go through the whole medical curriculum, pass their examinations honestly, and undertake the arduous and poorly paid labour of all-round general medicine.

But all such practice is detrimental to the public interests, injurious to medical progress, and knavish in itself. It ought, therefore, to be put a stop to; and the most effectual method of putting a stop to it is for medical men so to conduct their business as to convince the public that qualified practice is on the whole more convenient, more successful, and more generally satisfactory than unqualified. In other words, legitimate medicine must so far accommodate itself to the needs and moods of the public as to convince the whole community that, all things considered, competence and honesty in things medical are more deserving of patronage than incompetence and dishonesty.

The "pay ward" question in essence amounts to this, that certain advantages can be secured in hospitals which cannot be obtained outside them, except at a cost which is ruinous to all but the moderately rich. These advantages are of the class that concern health and life; and as health and life are among the most valued of all human possessions, it is but natural to expect that human nature will secure these possessions if possible—by paying for them if it can, but by getting them without payment if it cannot. That is the "pay question" in a nutshell. The medical part of that question is this: Can doctors so arrange the whole of the medical business of the nation among themselves that those who are able to pay doctors shall do so? Or must public charity be invoked to secure for people of modest means the superior advice and treatment which they could and would pay for if doctors had the business capacity to meet their legitimate demands in this regard?

It may be laid down as an axiom that people who are dangerously ill will leave no stone unturned to secure for themselves the best means of cure. Who shall blame them for this? Doctors themselves would be the very first to do the same in the same circumstances. With this, therefore, we must lay our account. If we are to survive and to make progress we must meet actual public needs.

Pay wards at hospitals have, to a certain extent, met the public needs we have indicated; and they have consequently become decidedly popular with the public. That they are injurious to private medical practice is certain: but that they will continue popular and become increasingly aggressive is also certain, unless the medical profession can do the only thing which will be effectual to check their growth, namely, substitute something better and more convenient for them.

What is the alternative to the pay ward system? The home hospital, the medical hotel, the nursing home; call it what we will. What the profession has to do is to put before the average man and woman of the middle class a comfortable home, in which he can live and be nursed and have the highest medical skill, on terms which are within his means. It may be answered that very few individual medical men have the capital necessary to start schemes of this kind. It is not desirable that they should do so; it is not a thing to be wished for that a few rich medical men should, by virtue of the possession of capital, swallow up all the practice, and leave their poorer brethren with no work to do. Medical men must combine, must become shareholders in co-operative medical undertakings. This is the new departure which holds out a promise of the solution of most of their difficulties.

Specialism has proved itself a rock upon which a great deal of practice profitable to the family practitioner has foundered during the past few decades. What is to be done with it? It must be captured and turned into a servant by the co-operation of general practitioners. The specialist, like the chemist and the dentist, must be made the operative hand of the legitimate physician, surgeon, and family practitioner. That this will be the natural development and ultimate future of specialism we need not for a moment doubt. The days of its primacy are already on the wane. The day of its modest and proper obedience and subordination to general medicine is close at hand. When medical men combine among themselves with the object of securing for their patients the best lodgings, the best nursing, the best general and the best special science and skill which the times afford; then, and not till then, will they strike a blow at incompetence within the profession and at knavery without it, from which there will be no recovery.

